# A Longitudinal Examination between Chronotype and Insomnia in Youths: A Cross-Lagged Panel Analysis

**DOI:** 10.3390/clockssleep6040037

**Published:** 2024-10-10

**Authors:** Forrest Tin Wai Cheung, Hao Fong Sit, Xiao Li, Joey Wing Yan Chan, Ngan Yin Chan, Yun Kwok Wing, Shirley Xin Li

**Affiliations:** 1 Sleep Research Clinic and Laboratory, Department of Psychology, The University of Hong Kong, Hong Kong; forrestc@connect.hku.hk (F.T.W.C.); hfsit@connect.hku.hk (H.F.S.); lixiaozq@hku.hk (X.L.); 2 Li Chiu Kong Family Sleep Assessment Unit, Department of Psychiatry, Faculty of Medicine, The Chinese University of Hong Kong, Hong Kong; joeywychan@cuhk.edu.hk (J.W.Y.C.); rachel.chan@cuhk.edu.hk (N.Y.C.); ykwing@cuhk.edu.hk (Y.K.W.); 3 The State Key Laboratory of Brain and Cognitive Sciences, The University of Hong Kong, Hong Kong

**Keywords:** insomnia, chronotype, eveningness, youths, longitudinal study

## Abstract

Adolescence and young adulthood are transitional periods associated with significant changes and challenges, leading to a heightened vulnerability to sleep disturbances and mental health difficulties. This stage is often associated with an increased preference for eveningness, manifested as a later chronotype. The current study aimed to investigate the directionality of the association between chronotype, based on an individual’s sleep–wake behaviour, and insomnia in young people using a two-wave panel design with a 12-month interval. A total of 370 participants aged 15–24 (mean age: 21.0 ± 2.0, 72.7% female) were recruited from local secondary schools and universities. Insomnia symptoms were assessed using the Insomnia Severity Index, while chronotype was measured using the Munich Chronotype Questionnaire. Temporal associations were analysed using a series of cross-lagged panel models. The best fitting and most parsimonious model indicated that a later chronotype at baseline predicts more severe insomnia symptoms at the 12-month follow-up after accounting for autoregressive effects. However, the opposite causal model, where baseline insomnia symptoms predicted the chronotype at the 12-month follow-up, was not supported. These findings suggest that a late chronotype may be a potential risk factor for the development of insomnia in young people, emphasising the importance of considering circadian factors in the prevention and treatment of sleep disturbances among this population.

## 1. Introduction

Adolescence and young adulthood represent a critical transitional period. Individuals at this stage undergo various biological and psychosocial changes, making them vulnerable to sleep and mental health difficulties [1]. A prominent change among youths is the intrinsic circadian phase delay and the shift in circadian preference towards eveningness, resulting in delayed bedtimes and rise times [2,3]. One previous cross-sectional study estimated that the prevalence of an evening chronotype, i.e., a preference for rest and activity later in the day, was 9.2–21.5% in youths aged 12–24, with girls being more morning-oriented than boys [4]. A recent meta-analytic study revealed that the evening chronotype was associated with increased risks of mental health problems, including general psychopathologies, mood-related disturbances, and anxiety problems in youths [5]. Meanwhile, sleep disturbance, especially insomnia, is also common in this young population, with a prevalence of up to 51% [6,7]. Female sex has been identified as one of the major demographic risk factors for insomnia, where females were found to be 1.41 times more likely than males to have insomnia [8]. The emergence of this sex difference coincides with the onset of puberty. Significant sex differences in the prevalence of insomnia become apparent during the later stages of puberty. Specifically, there was an overall 3.6-fold increase in insomnia symptoms from the start to the late stage of puberty for girls and a 2.1-fold increase for boys [1]. When left untreated, insomnia is often associated with adverse outcomes, such as major mental disorders (e.g., depression, bipolar disorder, psychotic disorder) [9]. Insomnia symptoms and eveningness often co-occur, with approximately half of the adolescents with insomnia being categorised as evening-type [10]. The existing literature, supported by cross-sectional and longitudinal studies, generally suggests that both eveningness and insomnia can independently contribute to poor daytime functioning and the risk of mental health issues [11,12].

Despite the comorbid relationship and common negative consequences shared by eveningness and insomnia, there has been limited research that delineates the relationship between chronotype and insomnia among the young population. On the one hand, eveningness may develop into insomnia due to a misalignment with one’s circadian phase, leading to sleep difficulties [13]. On the other hand, insomnia may contribute to delayed bedtime due to maladaptive coping behaviours, such as excessive use of electronic devices in bed [14]. Several cross-sectional studies found that an eveningness preference was associated with increased insomnia symptoms in youths [15,16]. For instance, a nationwide epidemiological study conducted in high school students in Korea found that individuals with an eveningness tendency had a 2.51-fold risk of having insomnia compared to their non-evening counterparts [16]. However, a causal relationship between chronotype and insomnia remained unclear due to the limitations of the cross-sectional design of these studies. Only very few longitudinal studies have explored the relationship between chronotype and insomnia in the context of mental and sleep health. For instance, Lin and colleagues conducted a survey study involving 1791 young adults (mean age = 27.2) and found that insomnia symptoms appeared to be a potential mediator in the relationship between evening chronotype and mental health and behavioural problems in young adults [17]. In a three-wave longitudinal study spanning from December 2019 to June 2020, which examined mental health and sleep among 831 adolescents (aged 14–19) during the COVID-19 pandemic, it was found that eveningness was associated with insomnia severity across all waves of assessments [18]. However, a longitudinal analysis of directionality between the two constructs was not explored in previous studies. Overall, the existing research on chronotype and insomnia primarily employs cross-sectional designs, which cannot ascertain the temporal relationship between the two conditions. While there have been some longitudinal studies, they typically focused on the relationship between these conditions and other outcomes (e.g., insomnia as a mediator between chronotype and mood-related outcomes). Additionally, previous longitudinal studies often used analytical methods, such as ordinary regression analyses, which failed to account for the reciprocal linkages between the variables over time.

A longitudinal panel approach could offer more comprehensive insights into the temporal relationship between the variables. The cross-lagged panel model (CLPM), which incorporates measures from baseline and follow-up in a single model, could facilitate the assessment of reciprocal relationships between variables across time [19]. This approach can address the development and mutual influence of chronotype and insomnia, thus providing more in-depth insights into the temporal relationship and potential reciprocal associations between the two constructs. 

Insomnia and eveningness are prevalent among youths, yet most of the existing research was focused on the adult populations. Given the high comorbidity of eveningness and insomnia, understanding their linkage from a longitudinal perspective could shed light on the aetiology of sleep problems in youths and potentially inform the future development of prevention and interventions for this vulnerable group. Considering these research gaps, we aimed to examine the directionality of the temporal association between chronotype and insomnia in youths using a two-wave design with a 12-month time interval.

## 2. Results

### 2.1. Descriptive and Correlation Analyses

There were no significant differences in age, sex, and chronotype between participants who completed the follow-up and those who did not. However, individuals who did not complete the follow-up survey at T2 had significantly more severe baseline insomnia symptoms (completers: 8.09 ± 4.72 vs. non-completers: 9.32 ± 5.21, *p* < 0.001) and had a higher proportion of having a past medical history (completers: 7.0% vs. non-completers: 13.4%, *p* = 0.003) compared to follow-up completers. A comparison between individuals who completed and did not complete the 12-month follow-up is presented in Supplementary Data Table S1. Out of the 370 included participants, 72.7% (269/370) were female, and the overall mean age at baseline was 21.08 ± 2.0 years. 

Descriptive statistics for insomnia severity, chronotype, sleep and wake time, and sleep duration derived from the MCTQ are presented in Table 1. Compared to T1, participants exhibited a significantly later chronotype (t = −2.41, *p* = 0.016) and wake time during free days (t = −2.50, *p* = 0.013) and workdays (t = −5.26, *p* < 0.001), as well as longer sleep duration during workdays (t = −3.15, *p* = 0.002) at T2. Insomnia severity and sleep time during free days and workdays did not differ significantly between T1 and T2 (*p* > 0.05). Insomnia severity based on the total ISI scores showed that 49.5% of respondents reported no insomnia (ISI score ≤ 7), 39.5% showed subthreshold insomnia (ISI score 8–14), 10.3% showed moderate clinical insomnia (ISI score 15–21), and 0.8% showed severe clinical insomnia (ISI score ≥ 22). In addition, the proportion of respondents that reported moderate or above severity in difficulty falling asleep, difficulty maintaining sleep, and early morning awakening was 28.1%, 21.4%, and 23.5%, respectively. Insomnia symptoms and chronotype at both time points were all significantly correlated (*p* < 0.01) except between insomnia symptoms at T1 and chronotype at T2 (Table 2).

### 2.2. Prevalence and Incidence of Insomnia

The prevalence of clinically significant insomnia (ISI > 14) was 14.0% at T1 and 9.6% at T2. Among those with insomnia at T1, 4.3% continued to report persistent insomnia at the 12-month follow-up. Nineteen participants developed insomnia during the 12-month follow-up period, resulting in a cumulative incidence of 2.64%.

### 2.3. Cross-Lagged Model of Insomnia and Chronotype

**Figure 1:**
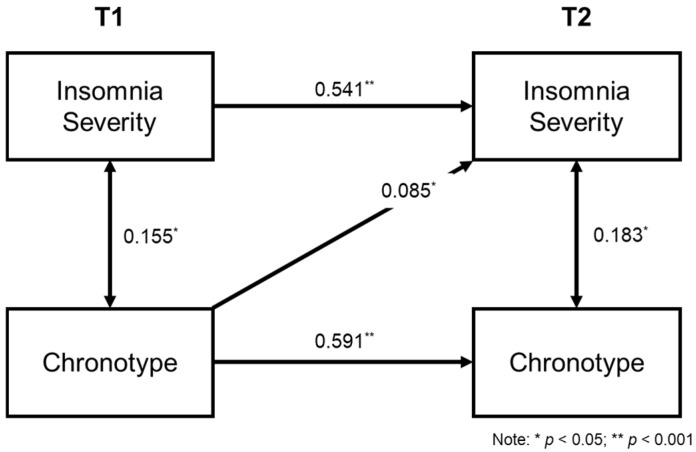
Standardised path coefficients from the cross-lagged panel model. Age and sex were added in model as control variables but not depicted in figure.

Fit indices for all competing models and chi-square difference tests are available in Table 3. *Model 3b*, the reversed causation model which tested the lagged association from T1 chronotype to T2 insomnia was selected as the best fitting and parsimonious model (χ^2^ (2) = 0.13, *p* = 0.937, CFI = 1.00, RMSEA = 0.000, SRMR = 0.004, AIC = 8881.71; Figure 1). Based on the principle that parsimonious models are preferred over less parsimonious models, given that they could provide a similar level of prediction while using fewer parameters [20], the reciprocal model (*Model 4*) was not selected as the final model, as the chi-square difference tests between *Model 4* (AIC = 8883.60) and *Model 3b* (AIC = 8881.71) were insignificant (Δχ^2^ = 0.12, *p* = 0.734). In the final model (*Model 3b*), the autoregressive paths for insomnia and chronotype from T1 to T2 were moderately strong (insomnia: *β* = 0.54, se = 0.04, *p* < 0.001; chronotype: *β* = 0.59, se = 0.04, *p* < 0.001). The cross-lagged paths revealed a significant relationship between chronotype at T1 and insomnia severity in T2 (*β* = 0.09, se = 0.13, *p* = 0.050), suggesting that later chronotype (i.e., eveningness) at baseline predicted greater insomnia symptoms at the 12-month follow-up after accounting for the autoregressive effects. Both control variables (i.e., age and sex) were insignificant across insomnia and chronotype at both time points in the model (*p* > 0.05). The path diagrams for all remaining competing models are available in Supplementary Data Figures S1–S4. The path diagram of the selected model with control variables depicted is available in Supplementary Data Figure S5. 

## 3. Discussion

The current study examined the directionality of the association between chronotype and insomnia in youths using a two-wave panel design with a 12-month follow-up in a series of structural equation models. Our findings demonstrated that the best fitting model was that in which chronotype at baseline predicted greater insomnia symptoms at the 12-month follow-up after accounting for the autoregressive effects. In contrast, the opposite causation model, where baseline insomnia symptoms were predictive of chronotype at the 12-month follow-up, had a poor fit, suggesting a unidirectional, rather than bidirectional, association between the two constructs.

Our results aligned with previous research, which suggested a strong comorbidity between eveningness and insomnia, particularly in youths [10]. The current study also extended the existing research by establishing a temporal association between chronotype and insomnia symptoms. The cross-lagged panel model suggested that a later chronotype predicts increased insomnia symptoms over time. However, it should be noted that the follow-up period (T2) took place during the COVID-19 pandemic (between April and December 2020). Comparison of the sleep characteristics between the two waves revealed a significant shift towards later wake times during workdays (T1: 08:10 vs. T2: 08:40, *p* < 0.001) and longer sleep durations (T1: 6.70 vs. T2: 7.03, *p* = 0.002). This pattern could potentially be attributed to lifestyle changes during the peak of the pandemic, such as remote schooling and working, which allowed for more flexible sleep schedules. Additionally, there was a small, albeit insignificant, decrease in insomnia symptoms from T1 to T2 (T1: 8.09 vs. T2: 7.68, *p* = 0.080). Therefore, the temporal association between chronotype and insomnia symptoms could be alternatively interpreted as, in the context of the COVID-19 pandemic, that later chronotype predicted less of a decrease in insomnia symptoms. It is worth noting that individuals would be subjected to the sleep constraints imposed by typical work and school schedules in non-pandemic times. Future research should aim to replicate these findings in a non-pandemic context to further elucidate the temporal relationship between chronotype and insomnia symptoms and to ensure the generalisability of the findings.

In terms of the directionality in which eveningness leads to more insomnia symptoms, one potential explanation is that youths with eveningness often have social obligations (e.g., morning classes at school, Sunday church services, and extracurricular activities) that require them to wake earlier than their endogenous circadian rhythm. Consequently, they may attempt to initiate sleep earlier to achieve the desired sleep duration. However, this conflicts with their circadian rhythm and potentially results in attempts to initiate sleep within or too close to their wake maintenance zone (WMZ; i.e., a period characterised by heightened core body temperature and alertness, typically 2–3 h before evening melatonin onset [21]), leading to difficulties in falling asleep and the manifestation of insomnia symptoms [13]. The role of the endogenous circadian rhythm in insomnia is corroborated by previous research demonstrating that 62% of individuals with insomnia had delayed core body temperatures, causing them to attempt sleep close to their WMZ [22]. Wright and colleagues also supported this notion with their finding that around 55% of adults with insomnia exhibited a late (i.e., >2 h) endogenous melatonin rhythm when measured under rigorous laboratory conditions [23]. Nonetheless, the relationship between insomnia and eveningness may be complicated by different factors. For example, certain sleep habits, such as bedtime procrastination and daytime napping, could also contribute to poor sleep quality and the experience of insomnia [24]. As a result, while it is possible that individuals with eveningness experience insomnia due to a misalignment of their endogenous circadian rhythm, it is important to recognise that other mechanisms could potentially contribute to this relationship. Further understanding of the sleep habits and characteristics of insomnia is needed to fully understand the associations between evening chronotype and insomnia. Nevertheless, the recurring difficulty in initiating sleep, which 28.1% of the sample complained of having moderate or above severity, due to the mismatch between earlier bedtimes and late circadian rhythm may result in frustration, worry, and maladaptive coping behaviours (e.g., daytime napping) [25], which can further perpetuate insomnia and contribute to a chronic course.

The opposite direction, where baseline insomnia predicted eveningness at 12 months, was not supported in the current analysis. One possible explanation is that individuals with insomnia tend to exhibit significant night-to-night variability in their sleep patterns [26]. While it is conceivable that their sleep onset time may be delayed at night when they experience difficulties initiating sleep, the manifestation of eveningness in this context can only be regarded as temporary on nights when the individuals have difficulties initiating sleep. In summary, the current evidence emphasised that chronotype, particularly eveningness, is a distinct factor contributing to the development of insomnia. Consequently, these findings suggest that greater attention to circadian factors is warranted in the conceptualisation and clinical management of insomnia among young people.

### 3.1. Implications

Our results emphasised the potential importance of circadian rhythms, especially in relation to eveningness, in the aetiology of insomnia in youths. Previous studies have consistently found that adolescents with eveningness were more likely to experience insomnia symptoms than their non-evening counterparts (e.g., Li et al. [10]), suggesting the implications of circadian-related factors in the manifestation of insomnia symptoms. This circadian-related insomnia may represent a unique phenotype of insomnia in youths. Nonetheless, there has been limited research to investigate this clinical presentation and its underlying pathophysiological mechanism.

Based on our current findings, it is suggested that interventions addressing chronotype could be beneficial in reducing insomnia symptoms in youths. Notably, the Transdiagnostic Sleep and Circadian (TranS-C) intervention is a non-pharmacological treatment that incorporates both cognitive behavioural therapy for insomnia and circadian-related components to tackle both sleep and circadian disturbances [27]. It has been shown to be a promising intervention for enhancing sleep quality and advancing the circadian phase in young people. In a study comparing the efficacy of TranS-C versus psychoeducation in 176 youths aged 10 to 18, TranS-C was found to significantly improve self-report sleep quality and advance circadian preference compared to psychoeducation alone at post-intervention, with a small to moderate effect [28]. Meanwhile, there has been a growing interest in bright light therapy (BLT) as an alternative, low-cost, and easily administered treatment strategy for improving sleep and addressing circadian factors in individuals with insomnia. The efficacy of using BLT in treating individuals with insomnia and eveningness was demonstrated in a meta-analysis of 53 studies, which showed that morning bright light exposure could significantly improve insomnia severity and circadian outcomes in adults [29]. Similarly, previous research also demonstrated that BLT effectively advanced the circadian phase, reduced sleep onset latency, and improved sleep duration in the adolescent population [30,31]. Overall, the current findings provide support to the notion that additional work on circadian factors could potentially benefit the treatment of insomnia in young individuals.

### 3.2. Limitations

Several limitations should be considered when interpreting the findings of our study. Firstly, the follow-up of this study was conducted during the COVID-19 pandemic. Research has shown that the COVID-19 pandemic led to changes in sleep patterns and an increase in insomnia symptoms, especially during periods of school suspension and social distancing [32,33]. Consequently, it would be beneficial for future research to replicate these findings within a non-pandemic context. In terms of the study sample, the age range of our participants, which spanned from 15 to 24, should be considered. Past studies have found that eveningness tendency peaks at age 20, followed by a period of stability in early adulthood [4,34]. As such, the participants in our study may have experienced a shift towards relative morningness as they matured from T1 to T2. Moreover, at T2, some of the older participants might experience environmental and psychosocial changes (e.g., transitioning from college to work) that are different from those of younger participants. In addition, our sample consisted predominantly of female participants (72.7%). Existing research has suggested that significant sex differences exist in both chronotype and insomnia, with more morningness and a higher risk of insomnia observed in females. To address this issue, we incorporated age and sex as controlled variables in our CLPM models. In terms of methodology, 55.4% of participants with significantly more severe insomnia symptoms were lost to follow-up, which could potentially indicate non-random attrition and possibly bias our results. Furthermore, our decision to use complete case analysis may have introduced bias and reduced the statistical power of our analysis. Therefore, caution should be exercised when interpreting our findings. Another limitation was our reliance on self-reported measures, which might be subject to recall bias. Future studies using ecological measures, such as a sleep diary, or objective measures, such as actigraphy or dim-light melatonin onset assessment, may provide more accurate assessments of sleep patterns and insomnia symptoms. Lastly, the current study focused solely on the relationship between chronotype and insomnia and did not include the measures that potentially confound the relationship between the two conditions, such as stress and depression severity. Future studies should consider investigating these potential moderators and mediators in the relationship between chronotype and insomnia.

## 4. Materials and Methods

### 4.1. Participants

**Figure 2:**
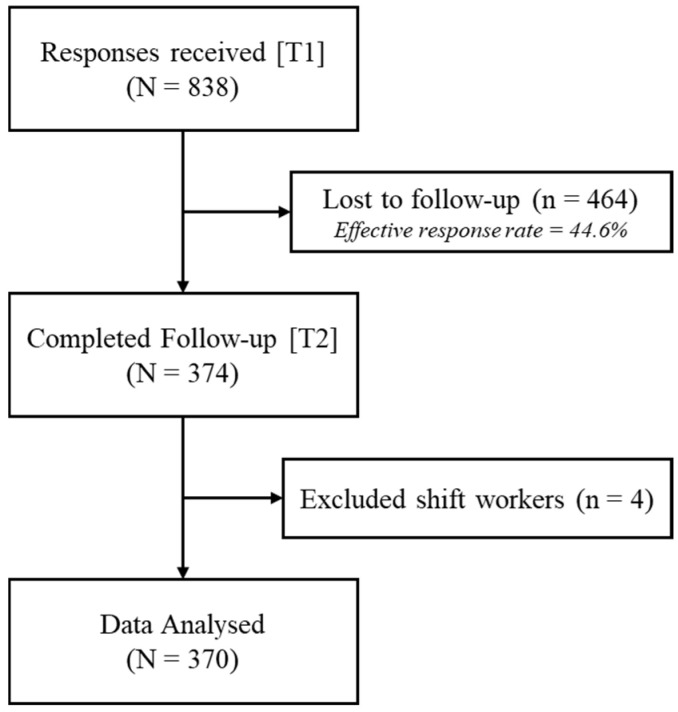
Study Flowchart.

Participants aged 15–24 were recruited from local secondary schools and universities in Hong Kong through social media promotions and email campaigns. This age range was chosen to broadly cover the developmental period from adolescence to the early phase of young adulthood, where insomnia is highly prevalent and accompanied by a shift in delayed sleep timing. The only criterion for exclusion is being a shift worker, as defined by having no or limited sleep opportunities during usual nighttime. The present study consists of two waves of data collection. The initial wave (T1) took place between April and October 2019, during which a total of 838 responses were received. The second wave (T2) occurred 12 months after the baseline, between April and December 2020, yielding a total of 374 responses (response rate = 44.6%). The second wave was slightly longer due to a delay in data collection at follow-up. Four cases that indicated they were shift workers were then removed, leaving a total of 370 samples included in the analysis. As suggested by previous research, a sample size of 200 or above was considered sufficient to conduct CLPMs with two waves [35]. The study flowchart is presented in Figure 2.

### 4.2. Procedure

At each wave, participants completed a set of questionnaires that included the measure of insomnia symptoms and the measure of sleep patterns and chronotype. Additionally, demographic information (e.g., age and sex) and self-reported past medical history were collected at baseline. To assess the participants’ past medical history, the following question was asked: ‘Have you been diagnosed with any medical or psychiatric disorder by a medical professional?’. Participants were then asked to indicate a binary response of either yes or no. 

All participants were provided with a consent form outlining the study’s purpose, methodology, and their rights as research participants before the start of the survey. Participants were then required to acknowledge their understanding and agreement to participate by ticking a checkbox once they had thoroughly reviewed the information. The ethical approval for the study was obtained from the Departmental Research Ethics Committee of the Department of Psychology at the University of Hong Kong in April 2019. 

### 4.3. Measurements

#### 4.3.1. Measure of Insomnia Symptoms

The 7-item Insomnia Severity Index (ISI) is a widely used self-report scale to evaluate the overall symptom severity of insomnia [36]. Cumulative scores range from 0 to 28, with higher scores indicating more severe insomnia symptoms. A score of 15 and above on the ISI is suggestive of having clinically significant insomnia symptoms [36]. The scale has previously been validated in the youth population in the local context [37]. The internal consistency of ISI for the current sample was α = 0.86 and 0.85 for baseline and follow-up, respectively. 

#### 4.3.2. Measure of Chronotype

The Munich Chronotype Questionnaire (MCTQ) collects time variables (clock time, hh:mm) of sleep and wake times separately for workdays and free days [38]. The midpoint of sleep (MSFsc), adjusted for sleep debt accumulated during the workweek, is used to determine an individual’s chronotype, with higher values indicating a later chronotype (more eveningness). The MCTQ has previously been validated for good reliability and validity in the Chinese youths [39].

### 4.4. Statistical Analysis

First, descriptive analyses were performed. A paired sample t-test was used to compare the differences between variables in T1 and T2. Second, Pearson’s correlation was used to investigate the bivariate relationships between insomnia symptoms and chronotype at T1 and T2. Third, a series of competing cross-lagged panel models on the cross-sectional and longitudinal associations between insomnia and chronotype, while controlling for age and sex, were analysed using maximum likelihood estimation. *Model 1* was the autoregressive model, which examined the temporal stabilities of insomnia and chronotype. *Model 2* added the cross-sectional associations between insomnia and chronotype at both time points. *Model 3a* and *Model 3b* were the causal models that added the lagged associations from baseline insomnia to later chronotype (*Model 3a*) and the reverse from baseline chronotype to later insomnia (*Model 3b*). Lastly, *Model 4* was the reciprocal model with a bidirectional-lagged relationship between insomnia and chronotype. Although maximum likelihood estimation can handle missing data in CLPMs, the considerable attrition from T1 to T2 in the sample (>50%) could potentially introduce bias in the analysis. Consequently, the current CLPM was estimated with complete data only (n = 370). 

The goodness-of-fit for the competing models was considered based on the conventional cut-off criteria for fit indices (i.e., chi-square statistics ≥ 0.05, comparative fit index ≥ 0.90, root mean square error of approximation ≤ 0.05, and standardized root mean square residual ≤ 0.06) [40]. In addition, the Akaike information criterion index was used to compare the quality of the model. Model fit for progressing models (*Models 2–4*) was compared with the previous models using chi-square difference tests. All statistical analyses were completed on R (version 4.1.0), and the cross-lagged panel analysis was conducted using the lavaan package [41].

## 5. Conclusions

The present findings of this two-wave prospective study provide evidence for a temporal relationship between chronotype and insomnia symptoms in youths, where an evening chronotype predicts greater insomnia problems. This directional relationship suggested the potential role of circadian preference in the development of insomnia and highlighted the need to consider circadian factors in clinical intervention. Future research should aim to replicate these findings using objective measures of sleep and chronotype, explore other potential factors contributing to sleep disturbance in youths, and investigate potential moderators and mediators to optimise treatment approaches.

## Figures and Tables

**Table 1:** Descriptive statistics on demographic, sleep, and circadian measures.

	T1	T2	*p*
Demographic			
Sex, (n, % Female)	269 (72.7%)		-
Age	21.08 (2.00)	22.10 (2.00)	**<0.001**
Derived from MCTQ			
Sleep Time (hh:mm)			
Free days	02:04 (01:38)	02:08 (01:37)	0.310
Workdays	01:21 (01:16)	01:23 (01:27)	0.510
Wake Time (hh:mm)			
Free days	10:26 (01:59)	10:39 (01:48)	**0.013**
Workdays	08:10 (01:27)	08:40 (01:39)	**<0.001**
Sleep Duration (hours)			
Free days	8.09 (1.88)	8.03 (1.35)	0.540
Workdays	6.70 (1.80)	7.03 (1.44)	**0.002**
Chronotype (hh:mm)	05:38 (01:39)	05:48 (01:31)	**0.016**
Derived from ISI			
Insomnia symptoms	8.09 (4.72)	7.68 (4.82)	0.080

Data are in mean (standard deviation) unless otherwise specified. Abbreviations: ISI = Insomnia Severity Index; MCTQ = Munich Chronotype Questionnaire. Bold values denote statistical significance.

**Table 2:** Zero-order correlations between insomnia symptoms and chronotype.

	Age	T1 ISI	T1 MSFsc	T2 ISI	T2 MSFsc
T1 ISI	0.101	-			
T1 MSFsc	−0.093	0.143 **	-		
T2 ISI	0.102 *	0.561 **	0.156 **	-	
T2 MSFsc	−0.262 **	0.077	0.611 **	0.193 **	-

Abbreviations: ISI = Insomnia Severity Index; MSFsc = Midpoint of sleep (derived from the Munch Chronotype Questionnaire). * *p* < 0.05, ** *p* < 0.01.

**Table 3:** Fit indices and chi-square difference tests.

	Model Fit						Model Comparison	
Competing Models	$\chi^{2}$	*df*	CFI	AIC	RMSEA	SRMR	$\Delta \chi^{2}$	*p*-Value
[*Model 1*] Autoregressive model	12.86	4	0.975	8890.45	0.077	0.059		
[*Model 2*] Cross-Sectional model	3.90	3	0.997	8883.48	0.028	0.022		
Comparison: M2 vs. M1							8.97	**0.003**
[*Model 3a*] Causal model (Insomnia to Chronotype)	3.74	2	0.995	8885.33	0.049	0.021		
Comparison: M3a vs. M1							9.12	**0.010**
Comparison: M3a vs. M2							0.15	0.69
[*Model 3b*] Reversed causal model (Chronotype to Insomnia)	0.13	2	1.000	8881.71	0.000	0.004		
Comparison: M3b vs. M1							12.73	**0.002**
Comparison: M3b vs. M2							3.77	**0.052**
[*Model 4*] Reciprocal model	0.02	1	1.000	8883.60	0.000	0.001		
Comparison: M4 vs. M1							12.85	**0.005**
Comparison: M4 vs. M2							3.88	0.14
Comparison: M4 vs. M3a							3.73	**0.054**
Comparison: M4 vs. M3b							0.12	0.73

Abbreviations: AIC = Akaike’s Information Criterion; CFI = Comparative Fix Index; df = degree of freedom; RMSEA = Root Mean Square Error of Approximation; SRMR = Standardised Root Mean Squared Residual. Note: M refers to the respective models (e.g., M1 = Model 1). All models were controlled for age and sex. Bold values denote statistical significance.

## Data Availability

Data involved in the current analysis are available upon reasonable request to the corresponding author.

## Supplementary Materials

The following supporting information can be downloaded at: https://www.mdpi.com/article/10.3390/clockssleep6040037/s1.
