# Supplementary material for: A Longitudinal Examination between Chronotype and Insomnia in Youths: A Cross-Lagged Panel Analysis
Source: Clocks Sleep. 2024 Oct 10;6(4):557–67. doi: 10.3390/clockssleep6040037 (PMC11503325; doi:10.3390/clockssleep6040037)

Table S1. Comparison between individuals who completed and did not complete the 12-month follow-up.

	Completed the Follow-up	Did not complete the follow-up	t / Chi^2	p
	n = 374	n = 464		
Age	21.1 (2.01)	21.3 (2.01)	1.54	.124
Sex (female)	273 (73.0%)	321 (69.2%)	1.46	.227
Insomnia symptoms (ISI)	8.09 (4.72)	9.32 (5.21)	3.55	**<.001**
Chronotype (MCTQ)	05:38 (01:39)	05:40 (01:36)	0.32	.749
Current/past history of physical and mental disorder(s)	26 (7.0%)	62 (13.4%)	9.05	**.003**

Data is in mean (standard deviation) unless otherwise specified.
Abbreviations: ISI = Insomnia Severity Index; MCTQ = Munich Chronotype Questionnaire

Figure S1. Model 1, the autoregressive model

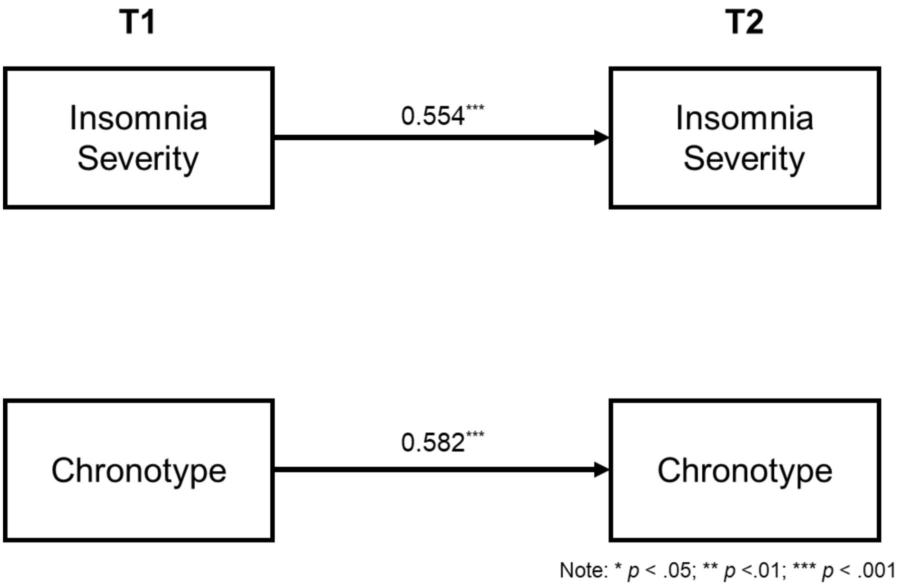

Figure S2. Model 2, the cross-sectional model

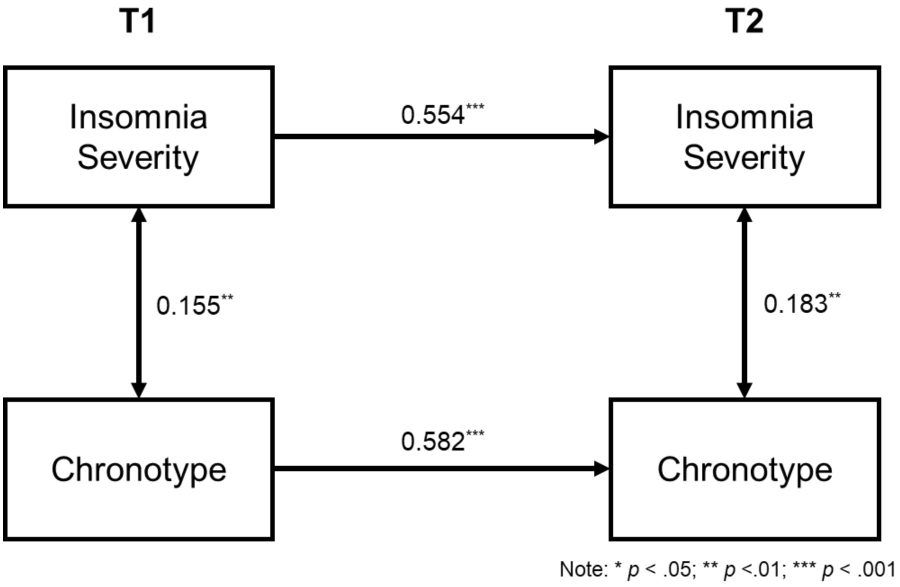

Figure S3. Model 3a, the causal model (insomnia > chronotype)

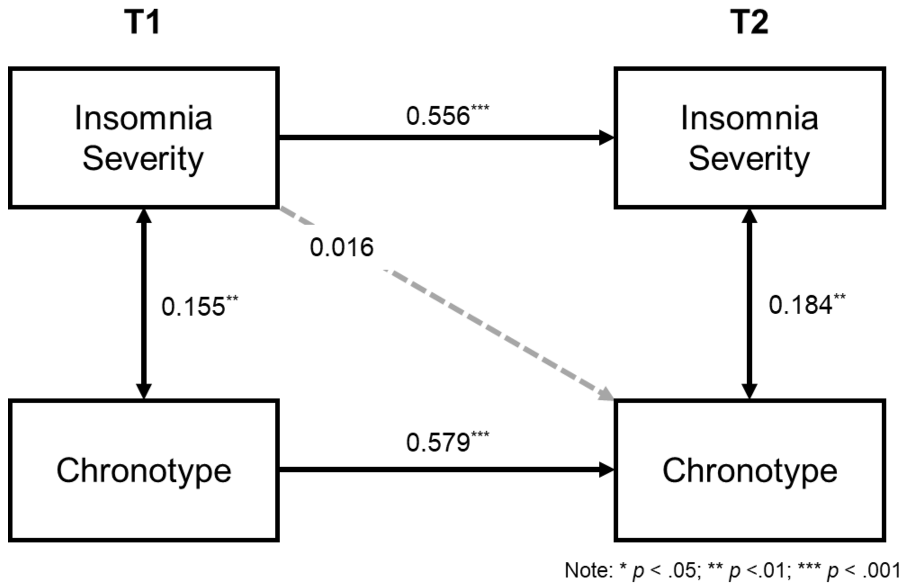

Figure S4. Model 4, the reciprocal model

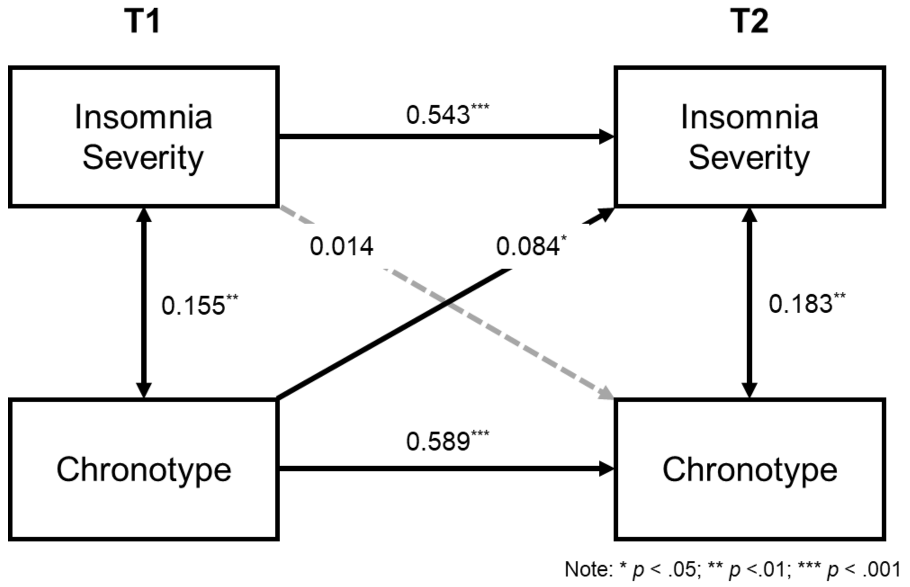

Figure S5. Model 3b, the causal model (chronotype > insomnia) with control variables (age and sex) depicted.

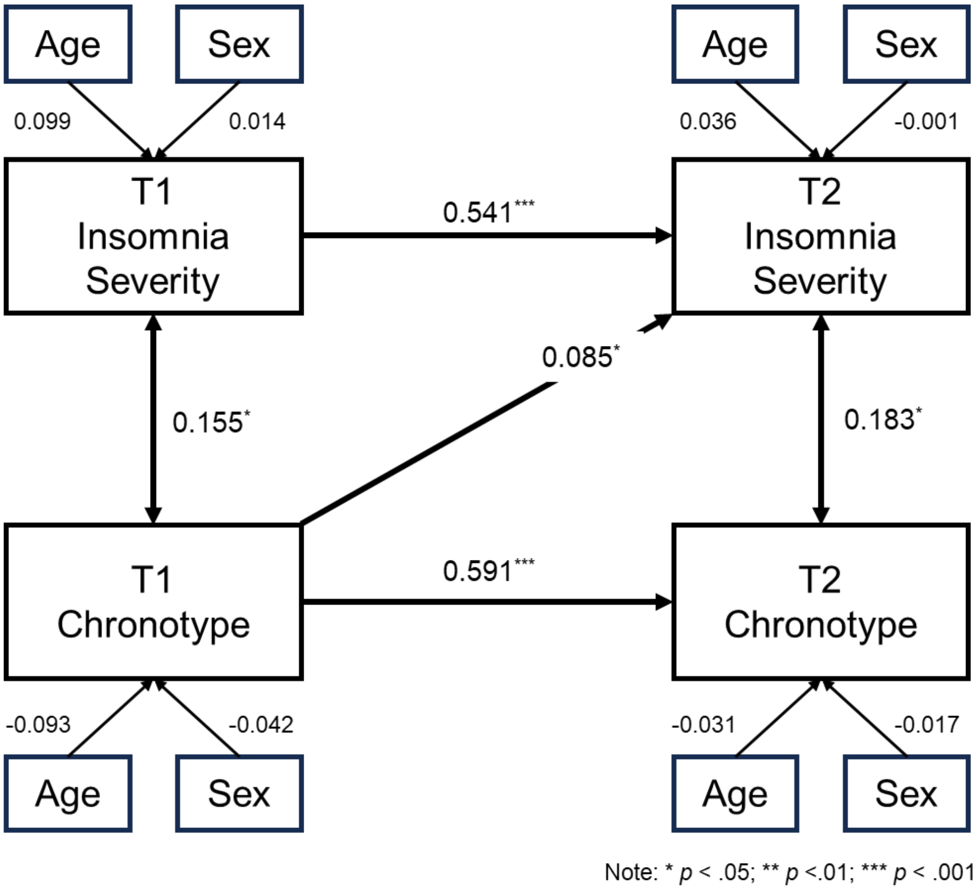

Supplement: Supplementary file 1 [file clockssleep-06-00037-s001.zip › clockssleep-3122214-supplementary.pdf]
